# ADAMTS-15 Has a Tumor Suppressor Role in Prostate Cancer

**DOI:** 10.3390/biom10050682

**Published:** 2020-04-28

**Authors:** Marley J. Binder, Scott McCoombe, Elizabeth D. Williams, Daniel R. McCulloch, Alister C. Ward

**Affiliations:** 1 School of Medicine, Deakin University, Waurn Ponds, VIC 3216, Australia; 2 Australian Prostate Cancer Research Centre–Queensland, Institute of Health and Biomedical Innovation, Queensland University of Technology (QUT), Translational Research Institute, Brisbane, QLD 4102, Australia; 3 Centre for Molecular and Medical Research, Deakin University, Waurn Ponds, VIC 3216, Australia

**Keywords:** ADAMTS, ECM, prostate cancer, tumor suppressor, VCAN, versikine

## Abstract

Extracellular matrix remodeling has emerged as an important factor in many cancers. Proteoglycans, including versican (VCAN), are regulated via cleavage by the proteolytic actions of A Disintegrin-like And Metalloproteinase domain with Thrombospondin-1 motif (ADAMTS) family members. Alterations in the balance between Proteoglycans and ADAMTS enzymes have been proposed to contribute to cancer progression. Here, we analyzed the expression of ADAMTS-15 in human prostate cancer, and investigated the effects of enforced expression in prostate cancer cell lines. ADAMTS-15 was found to be expressed in human prostate cancer biopsies with evidence of co-localization with VCAN and its bioactive cleavage fragment versikine. Enforced expression of ADAMTS-15, but not a catalytically-inactive version, decreased cell proliferation and migration of the ‘castrate-resistant’ PC3 prostate cancer cell line in vitro, with survival increased. Analysis of ‘androgen-responsive’ LNCaP prostate cancer cells in vivo in NOD/SCID mice revealed that ADAMTS-15 expression caused slower growing tumors, which resulted in increased survival. This was not observed in castrated mice or with cells expressing catalytically-inactive ADAMTS-15. Collectively, this research identifies the enzymatic function of ADAMTS-15 as having a tumor suppressor role in prostate cancer, possibly in concert with androgens, and that VCAN represents a likely key substrate, highlighting potential new options for the clinic.

## 1. Introduction

Prostate cancer represents the most common cancer-related morbidity affecting men in Western countries, including the United States [1]. Prostate cancer biology is complex, with the disease typically divided into two distinct stages. Initially ‘androgen-responsive,’ through expression of the androgen receptor (AR), over time a subset of these cells acquires the ability to proliferate without androgens [2]. During this so-called ‘castrate-resistant’ phase, that metastasis to secondary sites typically becomes clinically apparent, principally including bone, lung, liver, pleura and adrenal glands [2,3,4].

The extracellular matrix (ECM) has important functions in tissue architecture and function, forming a penetrable barrier that can be dynamically remodeled [5]. Altered ECM remodeling has been linked with unfavorable outcomes in several cancers, including melanoma, ovarian, cervical, breast, colon and prostate cancer [6,7,8,9,10,11]. The ECM is composed of multiple components, including numerous fibrous proteins and numerous proteoglycans (PGs) [5,12]. One of these PGs, versican (VCAN), has been shown to contribute to the loose hydrated structure of the ECM during key remodeling events, including those modulated in carcinogenesis, such as cell adhesion, migration, proliferation and angiogenesis [13].

VCAN has been shown to be up-regulated in prostate cancer along with the PGs syndecan-1, perlecan, decorin, biglycan, neural/glial antigen, serglycin and lumican [7]. Other studies have shown that increased VCAN is correlated with elevated prostate-specific antigen levels and poorer prognosis in prostate cancer, including an increased risk of metastasis following radical prostatectomy [14,15]. The V1 isoform of VCAN has been shown to promote prostate cancer cell motility [16,17], leading to decreased cell attachment to fibronectin coated substrates [15]. VCAN accumulation occurs in stromal tissue in prostate cancer, potentially mediated by androgens, and is an indicator of disease relapse in clinically localized prostate cancer [16,18].

VCAN is known to be regulated by members of the A Disintegrin-like And Metalloproteinase domain with Thrombospondin-1 motif (ADAMTS) family. ADAMTS-1, -4, -5, -9, -15 and -20 have been demonstrated to cleave VCAN [17,19,20,21]. This occurs within the GAG-β domain, forming a bioactive fragment termed versikine [19,22,23], which has been shown to play a role in immune regulation, such as in myeloma [24]. Numerous ADAMTS members have been implicated in tumorigenesis [25]. Of those that can cleave VCAN, ADAMTS-1 and -15 are the most highly expressed in prostate cancer cell lines [26], with ADAMTS-1 demonstrated to possess tumor suppressor activity in prostate cancer [27], and ADAMTS-15 expression shown to be androgen-dependent [28].

ADAMTS-15 acts as a tumor suppressor in breast and colorectal cancer [29,30]. Enforced expression of ADAMTS-15 was able to reduce motility of breast cancer cells and decrease angiogenesis independently of its catalytic activity. It additionally reduced breast cancer metastasis to the liver, although metastasis to the lung was increased [31]. Moreover, ADAMTS-15 expression positively correlated with improved prognosis in breast cancer patients [29,31]. Inactivating mutations in ADAMTS-15 have been identified in a subset of colorectal and pancreatic cancer patients [32,33]. Loss of ADAMTS-15 in colorectal cell lines resulted in increased tumor growth both in vitro and in vivo, which could be reversed by the re-introduction of ADAMTS-15, although the level of expression did not correlate significantly with cancer grade [30]. Collectively, this suggests a complex role for ADAMTS-15 in oncogenesis.

In this study, the role of ADAMTS-15 in prostate cancer was investigated. Expression of ADAMTS-15 was identified in human patient biopsies where it was co-localized with VCAN and its bioactive cleavage fragment versikine. Enforced expression of ADAMTS-15, impacted on proliferation, survival and migration of late-stage ‘castrate-resistant’ PC-3 prostate cancer cells in vitro, which was not reproduced with a catalytically-inactive mutant. ADAMTS-15 expression in early-stage ‘androgen-responsive’ LNCaP prostate cancer cells only impacted on survival in vitro but caused reduced tumorigenesis in NOD/SCID mice compared to those expressing the catalytically-inactive mutant, which was not observed in castrated mice. Together, these data indicate that ADAMTS-15 acts as a tumor suppressor in prostate cancer, likely through cleavage of VCAN to versikine, with its in vivo effects on early-stage prostate cancer cells influenced by androgens. This suggests that ADAMTS-15 represents a potential biomarker for prostate cancer, and that augmenting versican cleavage is a possible strategy for treatment.

## 2. Materials and Methods

### 2.1. Human Prostate Cancer Samples

Sections of human prostate at a variety of Gleason stages were obtained from the Australian Prostate Cancer BioResource, with the approval of the Deakin University Human Research Ethics Committee (Project #2010-104). 

### 2.2. Immunofluorescence

Immunofluorescence staining of human prostate cancer slides was performed as previously described [17]. Paraffin fixed tumor sections were blocked in normal goat serum and incubated with anti-ADAMTS-15 (Sapphire Biosciences, #AB45047), anti-VCAN GAG-β (Merk Millipore, Bayswater, Vic., Australia, #AB1033) and anti-V0/V1 Neo (versikine; Thermo Fisher Scientific, Scoresby, Vic. Australia, #PA1-1748A) diluted 1:200 in phosphate-buffered saline (PBS) containing 0.3% (v/v) Triton X-100 (PBST) (Astral), followed by goat anti-mouse Alexa Fluor 594 (Life Technologies, Mulgrave, Vic., Australia, #A11012) and counter-stained with DAPI vector shield (Abacus, #H-1200). Coverslips were sealed and allowed to dry before storing at 4 °C. Imaging was conducted on a Fluoview FV10I microscope using the Fluoview V2.1B imaging software package (Olympus, Notting Hill, Vic., Australia).

### 2.3. Cell Culture and Transfection

Lymph node adenocarcinoma of the prostate (LNCaP; ATCC CRL-1740) and prostate carcinoma derived-3 (PC-3; ATCC CRL-1435) cell lines were cultured in Dulbecco’s modified Eagle medium (DMEM) (Life Technologies) containing 10% (v/v) fetal bovine serum (FBS) (In Vitro Technologies, Noble Park, Vic., Australia) and 0.1% (w/v) penicillin/streptomycin (Pen/Strep) (Life Technologies) at 37 °C supplemented with 5% CO_2_. These cell lines were transfected using Lipofectamine (Life Technologies) with an empty pcDNA3.1MycHisA+ (Thermo Fisher Scientific) vector along with versions expressing mouse ADAMTS-15 (wild-type; OriGene) or ADAMTS-15EA (catalytically-inactive, E362A) [17] and clones selected in the presence of 700 µg/mL Geneticin (Life Technologies).

### 2.4. Western Blot

Lysates were prepared from cell clones using Protein lysis buffer (0.05 M Tris-HCL, pH 7.5, 0.001 M EDTA, 0.001 M EGTA, 10% (v/v) glycerol, 1% (v/v) Triton X-100, 0.05 M NaF, 0.005 M NaP_2_O_5_, 0.001 M Na_3_VO_4_) containing 1:20 protease inhibitor (Roche Diagnostics, North Ryde, NSW, Australia). Protein samples were separated by SDS-PAGE on a 7.5% gel and transferred to a PVDF membrane (VWR International, Tingalpa, Qld., Australia). The membrane was blocked in 5% (w/v) skim milk in Tris-buffered saline (0.05 M Tris, 0.15 M NaCl, pH 7.4) with 1% Tween 20 (TBST) for 1 h at room temperature with agitation, and incubated with either anti-Myc (Sigma Aldrich, Castle Hill, NSW, Australia, #M4439) or anti-GAPDH (Merk Millipore, #MAB374) at 1:200 dilution and washed with TBST before incubation with goat anti-mouse HRP (Abacus, Mitcham, Vic., Australia, #115-035-003). The membrane was washed six times with TBST and chemiluminescence detected using ECL prime (VWR International) using a Chemidoc apparatus (BioRad, Gladesville, NSW, Australia).

### 2.5. Migration Assay

Cells (~900,000) were aliquoted into six well plates in DMEM/10% (v/v) FBS and allowed to adhere for 12 h at 37 °C and 5% CO_2_ and migration assessed, as described [34]. A bent 200 µl pipette tip (Thermo Fisher Scientific) was used to ‘wound’ the cell monolayer before the media was removed and cells washed with serum-free DMEM media prior to addition of DMEM/2% (v/v) FBS. Images were taken at 0, 6 and 24 h using a light microscope (Leica Microsystems, North Ryde, NSW, Australia).

### 2.6. Proliferation Assay

Cells (~20,000) were aliquoted into a clear 96 well plate (VWR International) in DMEM/10% (v/v) FBS and incubated for 24 h and 48 h at 37 °C and 5% CO_2_. WST-1 reagent (Roche Diagnostics) was added to each well, incubated at 37 °C and 5% CO_2_ for 2 h, and absorbance at 450 nm measured using a Fusion-alpha HT plate reader (Perkin Elmer, Glen Waverley, Vic., Australia).

### 2.7. Apoptosis Assay

Cells (~900,000) were aliquoted into six well plates in DMEM/10% (v/v) FBS and allowed to adhere for 12 h at 37 °C and 5% CO_2_. Cells were treated with 0.1% (v/v) sodium azide (Sigma Aldrich) for 24 h and 72 h, respectively. Cells were then trypsinized and washed with 1 × PBS and apoptosis assessed using an Annexin V/PI kit (BD Bioscience, Macquarie Park, NSW, Australia) and analyzed on a FACSCANTO^TM^II (BD Bioscience).

### 2.8. Animal Methods

Castrated and intact NOD-SCID mice at 8–10 weeks old were obtained from the Animal Resource Centre and acclimatized for 2 weeks prior to experimentation. Mice were anaesthetized using isoflurane and injected subcutaneously into the left flank using a 26-gauge needle with 1 × 10^6^ LNCaP cells in equal volumes of PBS and Matrigel^®^ High Concentration (In Vitro Technologies, #354248). Mice had access to unlimited food and water at all times and were weighed daily following injection. Tumors were measured using calipers once visible, with tumor volume calculated by length × width^2^ × 0.5. Humane killing was performed via the CO_2_ method once tumors had reached 15 mm at the largest diameter or if significant (>10%) weight loss had occurred, or at 9 weeks for mice where no significant differences in survival compared with controls had been observed. All animal work was carried out with the approval of the Deakin University Animal Ethics Committee (Project #G18-2015) and in accordance with institutional guidelines.

### 2.9. Tumor Analysis

Mouse tumor samples were fixed in 4% (v/v) paraformaldehyde embedded in paraffin, subjected to routine processing, sectioned at 5 μm and mounted on slides. Tumor sections were stained with hematoxylin and eosin (Grale Scientific, Ringwood, Vic., Australia) before mounting with DPX (Sigma Aldrich) and imaging with an Axiocam HRC (Zeiss, North Ryde, NSW, Australia). Immunohistochemistry of mouse tumor sections was performed according to the manufacturer’s protocol. Briefly, deparaffinized tumor sections were incubated in hydrogen peroxide at room temperature for 10 min and washed twice with PBST before blocking with protein block. Slides were incubated with 1:200 of anti-Ki67 (Abcam, Melbourne, Vic., Australia, #AB15580) or anti-Caspase 3 (Abcam, #AB4051) followed by incubation with biotinylated goat anti-mouse or rabbit antibody as appropriate, and streptavidin-peroxidase and then developed with DAB chromogen. 

### 2.10. Statistical Analysis

Analysis for all experiments used GraphPad Prism software to determine statistical significance with student *t*-test, one-way ANOVA with Tukey’s post-hoc analysis and log-rank (Mantel-Cox) test, as appropriate. For immunofluorescence and immunohistochemistry, Image J software was used, utilizing the thresholding and intensity correlation tools [35]. Co-localization analysis used a plugin for Image J to determine Pearson’s Correlation Co-efficient and Mander’s Overlap [36]. 

## 3. Results

### 3.1. Localization of ADAMTS-15, VCAN and Versikine in Human Prostate Cancer Biopsies

**Figure 1:**
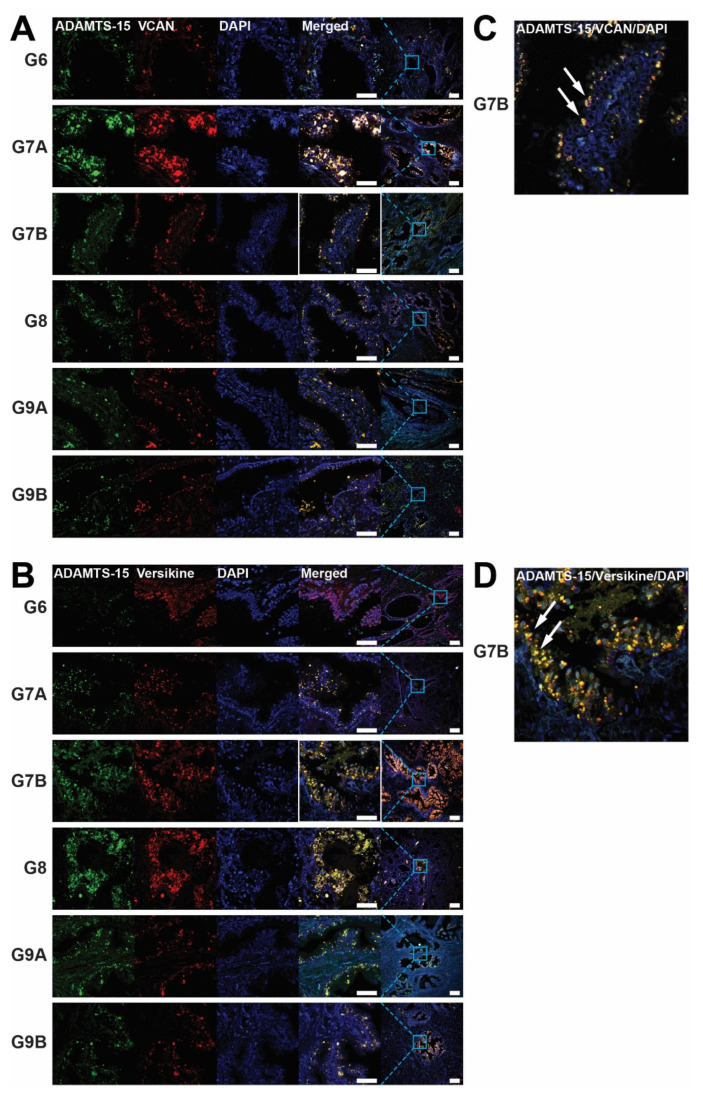
Expression of ADAMTS-15, versican and versikine in prostate cancer biopsies. (**A**,**B**) Immunofluorescence staining of ADAMTS-15 (green) and either versican (VCAN) (**A**) or versikine (**B**) (red) in concert with DAPI staining of nuclei (blue) in representative prostate cancer biopsies of the indicated Gleason grade. Single channel and merged images are provided at high magnification (scale bar 60 µm), with the merged image also shown at low magnification (scale bar 200 µm), with the relative position of the magnified area demarcated by the solid blue box. (**C**,**D**) Higher magnification of the merged images of ADAMTS-15/VCAN/DAPI (**C**) and ADAMTS-15/Verskine/DAPI (**D**) staining of G7B sections outlined by white boxes in panels **A** and **B**, respectively, with arrows indicating areas of co-localization in cells with an acinar epithelial morphology.

**Figure 2:**
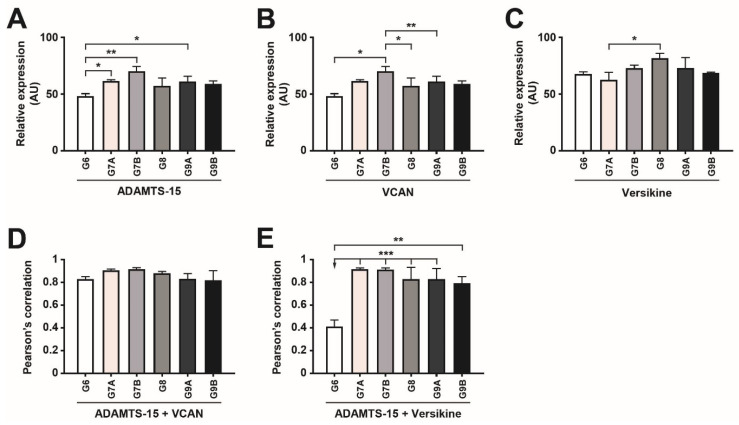
Quantification of expression and co-localization of ADAMTS-15, versican and versikine in prostate cancer biopsies. (**A**–**C**) Quantification of the relative area of expression of ADAMTS-15 (**A**), VCAN (**B**) and versikine (**C**) in the indicated Gleason grade showing mean and S.E.M. (* *p* < 0.05, ** *p* < 0.01, *** *p* < 0.001, n = 5 Gleason 6 = 3+3, n = 4 Gleason 8 = 4+4, 9A = 4+5 and 9B = 5+4, n = 3 Gleason 7A = 3+4 and 7B = 4+3). (**D**,**E**) Quantification of the co-localization of ADAMTS-15 with VCAN (**D**) and ADAMTS-15 with versikine (**E**) in the indicated Gleason grade prostate cancer biopsies as determined using Pearson’s Correlation Coefficient, showing mean and S.E.M. (** *p* < 0.01, *** *p* < 0.001, n = 5 Gleason 6 = 3+3, n = 4 Gleason 8 = 4+4, 9A = 4+5 and 9B = 5+4, n = 3 Gleason 7A = 3+4 and 7B = 4+3).

To explore the potential involvement of ADAMTS-15 in prostate cancer, biopsies of Gleason grade 6–9 tumors were subjected to immunofluorescence with antibodies directed to ADAMTS-15 along with its potential substrate VCAN (GAG-β) (Figure 1A) or the product of VCAN cleavage, versikine (Figure 1B). ADAMTS-15 expression was lowest in Gleason grade 6 biospecimens and significantly increased in grade 7A, 7B and 9A, being highest in grade 7B (Figure 1A,B and Figure 2A). By comparison, VCAN expression was also lowest in grade 6 and highest in grade 7B samples, the latter being significantly higher than grade 6, 8 and 9A (Figure 1A and Figure 2B). In contrast, versikine expression was most highly expressed in Gleason grade 8 samples, but this increase was only significant compared to grade 7A samples (Figure 1B and Figure 2C). Co-localization of ADAMTS-15 was apparent with both VCAN and versikine (Figure 1A–D). Quantification of this using Pearson’s Correlation Co-efficient confirmed strong co-localization of ADAMTS-15 and VCAN, which was highest in Gleason grade 7 samples (Figure 2D). Strong co-localization of ADAMTS-15 and versikine was also evident in all samples except grade 6, which showed significantly less co-localization compared to all other samples (Figure 2E). Similar results were obtained using Mander’s Overlap (Figure S1), except co-localization of ADAMTS-15 and VCAN in grade 7B reached statistical significance compared to grade 6 (Figure S1A). In all cases, expression was almost exclusively seen in acinar epithelial cells. Collectively, these data are generally consistent with ADAMTS-15 utilizing VCAN as a substrate to yield versikine in prostate cancer.

### 3.2. Enforced ADAMTS-15 Expression in Prostate Cancer Cell Lines

**Figure 3:**
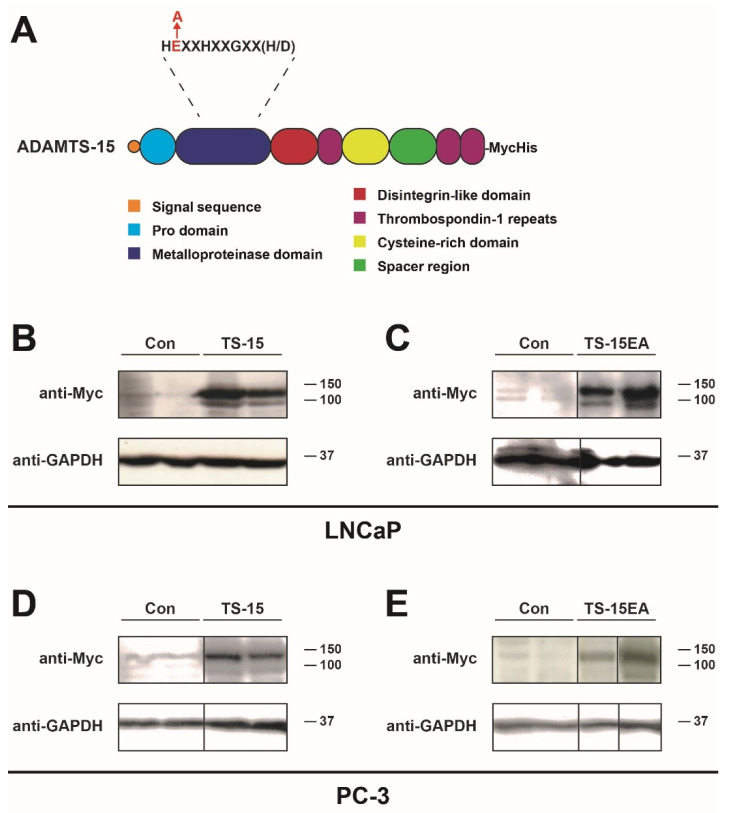
Enforced expression of ADAMTS-15 and ADAMTS-15EA in prostate cancer cell lines. (**A**) Structure of ADAMTS-15 and its catalytically inactive mutant, with domains identified in the key, the position of the inactivating E to A mutation in the metalloproteinase domain highlighted in red, and the C-terminal Myc/His tag indicated. (**B**–**D**) Western blot analysis of representative stable LNCaP (**B**,**C**) and PC-3 (**D**,**E**) transfectant clones as indicated containing empty pcDNA3.1 (Con) or pcDNA3.1 expressing ADAMTS-15 (TS-15) (**B**,**D**) or ADAMTS-15EA (TS-15EA) (**C**,**E**) with anti-Myc antibody to detect expression of tagged ADAMTS-15 and ADAMTS-15EA. Note the presence of zymogen (upper bands) and mature forms (lower bands) in each case. Anti-GAPDH was used as a loading control, and the relative position of size markers indicated.

To further investigate the role of ADAMTS-15 in prostate cancer, LNCaP and PC-3 cell lines were used as representative early-stage ‘androgen-responsive’ and late-stage ‘castrate-resistant’ prostate cancer cells, respectively [37]. These were transfected with pcDNA3.1 containing sequences encoding Myc/His-tagged wild-type ADAMTS-15 or catalytically-inactive ADAMTS-15EA or empty pcDNA3.1 vector as a control (Figure 3A). Clones were selected with neomycin and evaluated by Western blot analysis with anti-Myc to identify expressing clones, with GAPDH used as a loading control (Figure 3B–E). Both the zymogen and mature forms of Myc/His-tagged ADAMTS-15 and ADAMTS-15EA were observed at the expected molecular weight, with only weaker non-specific bands seen in pcDNA3.1 transfectants. A number of independently isolated clones expressing higher levels of the respective ADAMTS-15 isoforms were used for subsequent analyses. 

### 3.3. Effect of ADAMTS-15 Expression on Proliferation

**Figure 4:**
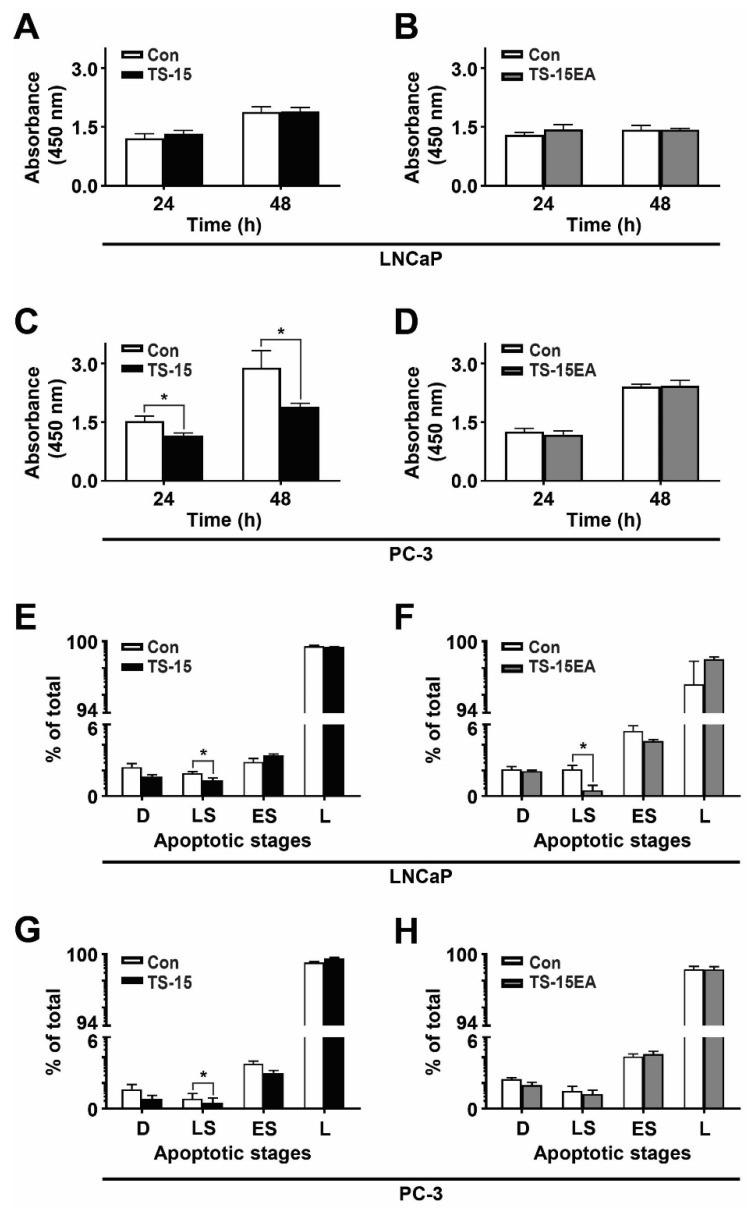
Proliferation and survival analysis of ADAMTS-15 and ADAMTS-15EA transfectants. (**A**–**D**) Relative proliferation of LNCaP cells expressing ADAMTS-15 (**A**) or ADAMTS-15EA (**B**), and PC-3 cells expressing ADAMTS-15 (TS-15) (**C**) or ADAMTS-15EA (TS-15EA) (**D**) in comparison to pcDNA3.1 controls (Con) at the times indicated using the WST-1 method and measuring absorbance at 450 nm (* *p* < 0.05; n = 3 pcDNA3.1, n = 4 ADAMTS-15 and ADAMTS-15EA). (**E**–**H**) Relative survival of LNCaP cells expressing ADAMTS-15 (TS-15) (**E**) or ADAMTS15EA (TS-15EA) (**F**), and PC-3 cells expressing ADAMTS-15 (**G**) or ADAMTS-15EA (**H**) compared to respective pcDNA3.1 controls (Con). Apoptosis was quantified by staining with FITC and PI and assessing cells in the four quadrants: D = dead cells, LS = late stage apoptosis, ES = early stage apoptosis, L = live cells (* *p* < 0.05; n = 3 pcDNA3, n = 4 ADAMTS-15 and ADAMTS-15EA).

The in vitro proliferation of individual clones was analyzed using the WST-1 assay. LNCaP cells expressing ADAMTS-15 (Figure 4A) or ADAMTS-15EA (Figure 4B) showed no significant difference compared to controls. However, PC-3 transfectants expressing ADAMTS-15 exhibited a large, statistically-significant decrease in proliferation at both 24 h and 48 h in comparison to pcDNA3.1 controls (Figure 4C). In contrast, no significant differences were observed in PC-3 cells expressing ADAMTS-15EA at 24 h and 48 h compared to pcDNA3.1 controls (Figure 4D). These results suggest a role for ADAMTS-15 in suppressing proliferation, particularly of late stage castrate-resistant prostate cancer cells that was dependent on its catalytic activity.

### 3.4. Effect of ADAMTS-15 Expression on Survival

The effects of ADAMTS-15 overexpression on cell survival was next investigated. A significant decrease in the number of late stage apoptotic cells was detected in LNCaP cells expressing ADAMTS-15 when compared to pcDNA3.1 controls (Figure 4E), which was also observed in LNCaP cells expressing ADAMTS-15EA (Figure 4F). PC-3 cells expressing ADAMTS-15 also showed significantly decreased late stage apoptotic cells compared to pcDNA3.1 controls (Figure 4G), but ADAMTS-15EA clones showed no difference compared to pcDNA3.1 controls (Figure 4H). This points to a small but significant pro-survival role for ADAMTS-15.

### 3.5. Effect of ADAMTS-15 Expression on Migration

**Figure 5:**
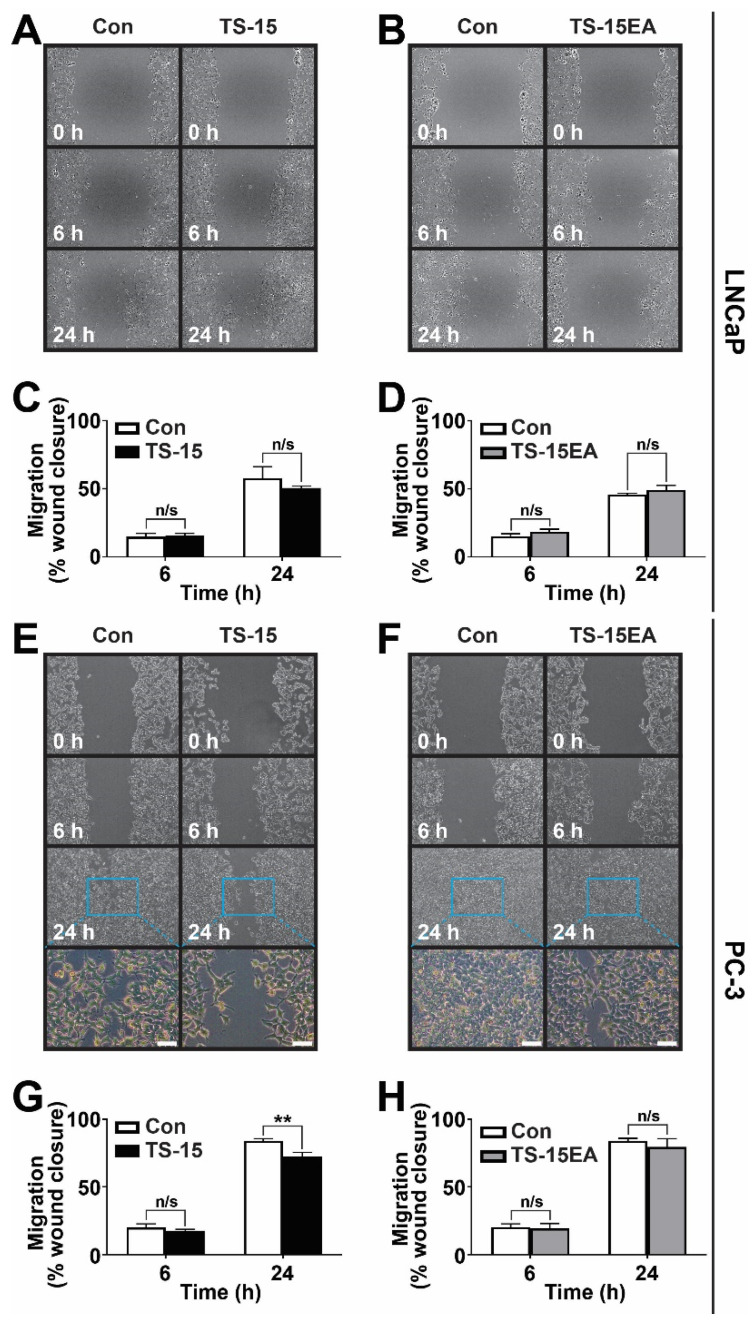
Migration analysis of ADAMTS-15 and ADAMTS15EA transfectants. Migration of LNCaP cells expressing ADAMTS-15 (**A**,**C**) and ADAMTS-15EA (**B**,**D**) or PC-3 cells expressing ADAMTS-15 (TS-15) (**E**,**G**) and ADAMTS-15EA (TS-15EA) (**F**,**H**) compared to pcDNA3.1 controls (Con) at the times indicated following wounding. Shown are representative images of cell migration (**A**,**C**,**E**,**F**), with the boxed areas in panels E and F shown at higher magnification immediately below (scale bar 100 μm). The percentage of migrating cells are shown (**C**,**D**,**G**,**H**) (** *p* < 0.01; n = 3 pcDNA3, n = 4 ADAMTS-15 and ADAMTS-15EA).

Migration was evaluated using the scratch ‘wound healing’ assay [34,38]. LNCaP cells expressing ADAMTS-15 (Figure 5A,C) or ADAMTS-15EA (Figure 5B,D) demonstrated no statistically significant difference in migration compared to pcDNA3.1 controls. By contrast, decreased cell migration was observed in PC-3 cells expressing ADAMTS-15 at 24 h post wounding when compared to pcDNA3.1 controls (Figure 5E,G). However, no significant differences were observed between PC-3 cells expressing ADAMTS-15EA and pcDNA3.1 controls (Figure 5F,H). No obvious differences in the morphology of migrating cells was observed (Figure 5E,F). Collectively, these results indicate a cell- and activity-dependent role for ADAMTS-15 in migration of late ‘castrate-resistant’ stage prostate cancer cells.

### 3.6. Effect of ADAMTS-15 Expression on Androgen-Responsive Tumor Growth In Vivo

**Figure 6:**
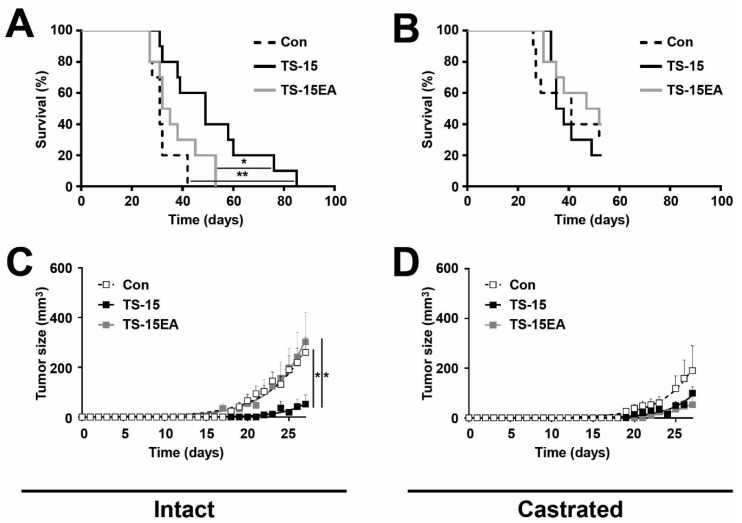
Tumorigenicity of androgen-responsive LNCaP xenografts in NOD/SCID mice. (**A**,**B**) Kaplan-Meier survival curves for intact (**A**) and castrated (**B**) male mice bearing LNCaP tumors expressing ADAMTS-15 (TS-15) or ADAMTS-15EA (TS-15EA) or pcDNA3.1 containing controls (Con), as indicated. Individual mice are shown, with statistical significance provided (* *p* < 0.05, ** *p* < 0.01 within mouse cohorts #*p* < 0.01 between mouse cohorts; log-rank Mantel-Cox test; n = 10 for intact, and n = 5–8 for castrated). (**C**,**D**) Growth of LNCaP tumors in intact (**C**) and castrated (**D**) mice from panels A and B, expressed as tumor size as estimated from external caliper measurements. These are shown as mean ± S.E.M. across all mice in each cohort, with statistical significance provided (* *p* < 0.05; Student’s *t* test; n = 10).

To gain further insight into the potential in vivo functions of ADAMTS-15, including its potential interaction with androgens, the early ‘androgen-responsive’ LNCaP cell lines stably expressing ADAMTS-15 or ADAMTS-15EA along with pcDNA3.1 containing control cell lines were analyzed in the NOD/SCID mouse xenograft model [39]. These experiments were performed in parallel on intact and castrated male mice to directly examine possible androgen-mediated effects. Intact male mice injected with LNCaP cells expressing ADAMTS-15 showed significantly increased survival compared to those injected with pcDNA3.1 containing control cells or those expressing ADAMTS-15EA (Figure 6A). This correlated with a significant decrease in tumor growth rate of those injected with ADAMTS-15 expressing LNCaP cells in comparison to those injected with pcDNA3.1 containing controls or ADAMTS-15EA expressing cells (Figure 6C). In contrast, mice injected with LNCaP cells expressing ADAMTS-15EA showed no significant difference in survival (Figure 6A) or tumor growth rate (Figure 6C) compared to those injected with pcDNA3.1 containing controls. This suggested that ADAMTS-15 exerts a protective function that was dependent on its catalytic activity. Castrated mice showed improved survival compared to intact mice following injection of pcDNA3.1 containing control cells or those expressing ADAMTS-15EA (Figure 6B), which correlated with reduced tumor growth (Figure 6D). However, castrated mice injected with LNCaP cells expressing ADAMTS-15 no longer showed a significant difference to those injected with pcDNA3.1 controls or ADAMTS-15EA expressing cells in terms of survival (Figure 6B) or tumor growth (Figure 6D). Tumors were harvested, weighed, sectioned and analyzed with respect to proliferation, as assessed with anti-Ki-67, and apoptosis, as determined using anti-Caspase 3. In comparison to tumors derived from control LNCaP cells, those derived from cells expressing ADAMTS-15 showed reduced size (Figure S2A) and relative growth rate (Figure S2B) as well as percentage of cells positive for Ki-67 staining but not anti-Caspase 3 staining (Figure S2C–D).This indicates that the in vivo protective effects of ADAMTS-15 on LNCaP cell tumorigenesis were likely influenced by androgens and affected proliferation.

## 4. Discussion

Approximately one in six men in developed countries will be diagnosed with prostate cancer in their lifetime [40]. Despite advances in early detection and treatment, this disease remains a major burden on society [41]. Emerging as a key player in the progression of numerous cancers is ECM regulation, particularly through the PG VCAN [5,13]. VCAN expression is known to be up-regulated during prostate cancer progression and is linked to poor prognosis [14,15,16,18]. ADAMTS proteases are multi-domain polypeptides involved in ECM regulation [31,42]. ADAMTS-1, -4, -5, -9, -15 and -20 are known to cleave VCAN [17,19,20,21], although non-catalytic roles have also been identified for ADAMTS enzymes [31,42,43,44]. Several of these family members also impact cancer progression [25,43,44,45,46], which prompted the examination of ADAMTS-15 in prostate cancer.

Increased accumulation of ADAMTS-15 was observed in epithelial tissue of high grade prostate tumors with the highest expression detected in Gleason grade 8 samples. Comparatively, the highest expression of VCAN was observed in Gleason grade 7 samples. Importantly, ADAMTS-15 and VCAN were found to be statistically co-localized in all grades, but was highest in Gleason grade 7 samples. This is interesting, as Gleason grade 7 is at the cusp between good and poor prognosis [47]. Of note, decreased VCAN expression was observed in Gleason grade 8 samples, in which maximum versikine expression was seen, with strong co-localization between ADAMTS-15 and versikine also observed. This is consistent with the hypothesis that ADAMTS-15 acts to cleave VCAN to form versikine in prostate cancer. VCAN and versikine can both bind hyaluronan (HA) [25,48], which represents a likely tether for these molecules in prostate cancer. The only caveat to these experiments is that the ADAMTS-15 antibody was directed to the molecule’s pro-peptide, and thus, may not exactly reflect the localization of the active enzyme.

To consider the potential for both catalytic and non-catalytic functions for ADAMTS-15, early ‘androgen-responsive’ stage LNCaP and late ‘castrate-resistant’ stage PC-3 prostate cancer cell lines were generated that stably expressed wild-type ADAMTS-15 or the catalytically-inactive ADAMTS-15 (ADAMTS-15EA) and characterized in vitro and in vivo. Overexpression of ADAMTS-15, but not ADAMTS-15EA, caused a significant decrease in cell proliferation and survival in late-stage PC-3 cells in vitro. This indicates that the metalloproteinase activity of ADAMTS-15 was required to mediate its negative impact on proliferation and survival. This result contrasted with a study in breast cancer in which ADAMTS-15 expression did not affect either proliferation or survival [31]. Expression of ADAMTS-15, but not ADAMTS-15EA, also significantly decreased migration of PC-3 cells. This effect was in agreement with the decreased migration observed for breast cancer cells expressing ADAMTS-15, although in this setting, the effect on migration was not dependent on the catalytic activity of ADAMTS-15 [31]. No effect was observed in proliferation of early-stage LNCaP cells expressing ADAMTS-15 or ADAMTS-15EA, but apoptosis was affected in both cases. No significant impact of ADAMTS-15 expression on migration of early-stage LNCaP cells was observed. LNCaP represent a less malignant stage of prostate cancer than PC-3 cells, with reduced levels of both proliferation and migration as well as lower VCAN expression (data not shown), which might explain why tumor-suppressor functions for ADAMTS-15 were less readily observed in this cell line. We did confirm that expression of ADAMTS-15 in this cell line induced VCAN cleavage (data not shown), confirming its functionality.

Morever, the ‘androgen-responsive’ LNCaP cells provided an opportunity to explore the potential interaction with androgens in vivo. Injection of LNCaP cells expressing ADAMTS-15 into intact mice resulted in significantly increased survival in comparison to those injected with LNCaP cells expressing ADAMTS-15EA or control cells (median survival of 49 days compared to 33.5 and 31 days, respectively). Furthermore, the growth rates of tumors formed from LNCaP cells expressing ADAMTS-15 was significantly reduced (Con: 0.058 ± 0.009 g/day; ADAMTS-15: 0.032 ± 0.006 g/day), while levels of the proliferation marker Ki-67 [49] in tumor sections showed revealed a similar decrease (Con: 25.6 ± 2.9%; ADAMTS-15: 18.8 ± 2.0%), whereas the apoptotic marker Caspase 3 [50] was not significantly altered. This was consistent with a previous study showing knock-down of ADAMTS-15 in colon cancer cells resulted in increased subcutaneous tumor growth in NOD/SCID mice [30]. Collectively, these results are indicative of a tumor suppressor role for ADAMTS-15 that is dependent on its catalytic activity. The reasons for the differences between in vitro and in vivo outcomes, particularly proliferation, remain unclear. We can only speculate on potential factors that are responsible, such as differences in ECM composition, interactions with other cells and the presence of cytokines, androgens and other external factors.

In fact, androgens are very important in prostate cancer biology, and thus, their potential importance was investigated by parallel injection of LNCaP clones into castrated mice, which have been shown to be able to support the growth of these cells [51]. In this setting mice injected with LNCaP cells expressing ADAMTS-15 showed no survival advantage when compared to those injected with cells expressing ADAMTS-15EA or pcDNA3.1 controls, with tumor growth rates no longer significantly different. This suggests that the in vivo tumor suppressor function of ADAMTS-15 in these cells is significantly influenced by androgens. Previous in vitro studies have shown that LNCaP cells express AR, with knockdown of AR resulting in significantly reduced growth and apoptosis [52], whereas elevated AR expression enhanced proliferation in these cells [53]. The interaction between androgens/AR and ADAMTS family members has not been well studied. However, ADAMTS-15 expression was shown to be negatively regulated by the androgen 5α-dihydrotestosterone in LNCaP cells [28]. Whether this is relevant to this study, where ADAMTS-15 expression was controlled by a heterologous promoter, remains to be seen. The clinical consequences of this mode of regulation are also difficult to predict, such that androgen deprivation therapy may serve to blunt the tumor suppressor functions of ADAMTS-15, the levels of which may separately become increased. However, in castrate-resistant cells—even those expressing AR, androgen-mediated signaling does not contribute to proliferation or migration [54]. Clearly, more research is required to delineate the relationship between the androgen/AR axis and ADAMTS-15.

## 5. Conclusions

We have shown that ADAMTS-15 influences important aspects of tumorigenesis, including proliferation, survival and migration. Furthermore, we have demonstrated the reliance of a functional metalloproteinase domain for these effects. Moreover, androgens appeared to impact on the tumor suppressor role of ADAMTS-15. Finally, co-localization of ADAMTS-15, VCAN and versikine suggests ADAMTS-15 regulates VCAN cleavage as a likely mechanism to impact on cancer progression. Other PGs may also play a role, particular those that are up-regulated in prostate cancer, such as syndecan-1, perlecan, decorin, biglycan, neural/glial antigen 2, serglycin and lumican [7]. Indeed, poor prognosis in prostate cancer has been linked to elevated biglycan [55] and syndecan-1 [56]. However, thus far, the only documented substrates for ADAMTS-15 are VCAN [57] and aggrecan [46], while in breast cancer, where the effects of ADAMTS-15 on cell migration were shown to involve syndecan-4, this was independent of its metalloproteinase activity [46]. Indeed, studies on a docetaxel-resistant PC-3 subline demonstrated that targeting VCAN impacted on both proliferation/viability [58], highlighting the importance of this substrate. Together, this suggests thqat ADAMTS-15 represents a potential biomarker for prostate cancer, and that augmenting versican cleavage is a possible strategy for treatment. Moreover, ADAMTS-1 is also able to function as a versicanase [25], with evidence that it is reduced in prostate cancer [27], suggesting it may act in concert with ADAMTS-15. Clearly further research is warranted to understand the interactions of ADAMTS family members within the complex ECM milieu.

## Supplementary Materials

The following are available online at https://www.mdpi.com/2218-273X/10/5/682/s1, Figure S1: Quantification of co-localization of ADAMTS-15 with VCAN and versikine in prostate cancer biopsies. Figure S2: Impact of ADAMTS-15 expression on tumor growth, proliferation and apoptosis.
