# Supplementary material for: ADAMTS-15 Has a Tumor Suppressor Role in Prostate Cancer
Source: Biomolecules. 2020 Apr 28;10(5):682. doi: 10.3390/biom10050682 (PMC7277637; doi:10.3390/biom10050682)
Supplement: Supplementary file 1 [file biomolecules-10-00682-s001.pdf]

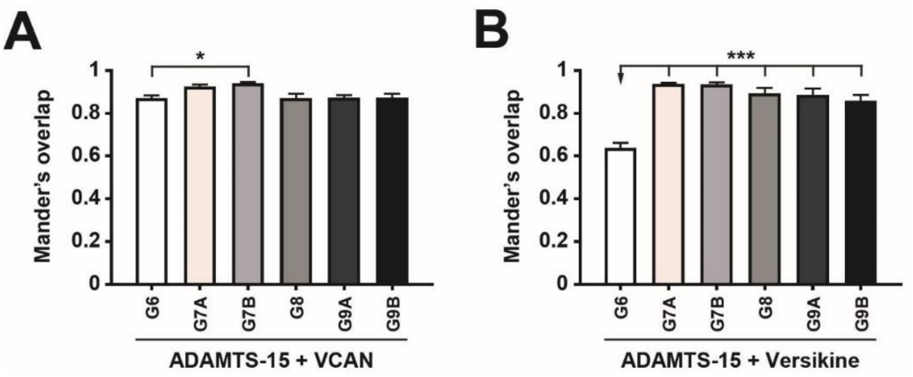

Supp. Figure 1. Quantification of co-localization of ADAMTS-15 with VCAN and versikine in prostate cancer biopsies. Extent of co-localization of ADAMTS-15 with VCAN **(A)** and ADAMTS-15 with versikine **(B)** in the indicated Gleason grade prostate cancer biopsies as determined using Mander's Overlap, showing mean and S.E.M. (*$p<0.05$, ***$p<0.001$, n=5 Gleason 6=3+3, n=4 Gleason 8=4+4, 9A=4+5 & 9B=5+4, n=3 Gleason 7A=3+4 & 7B=4+3).

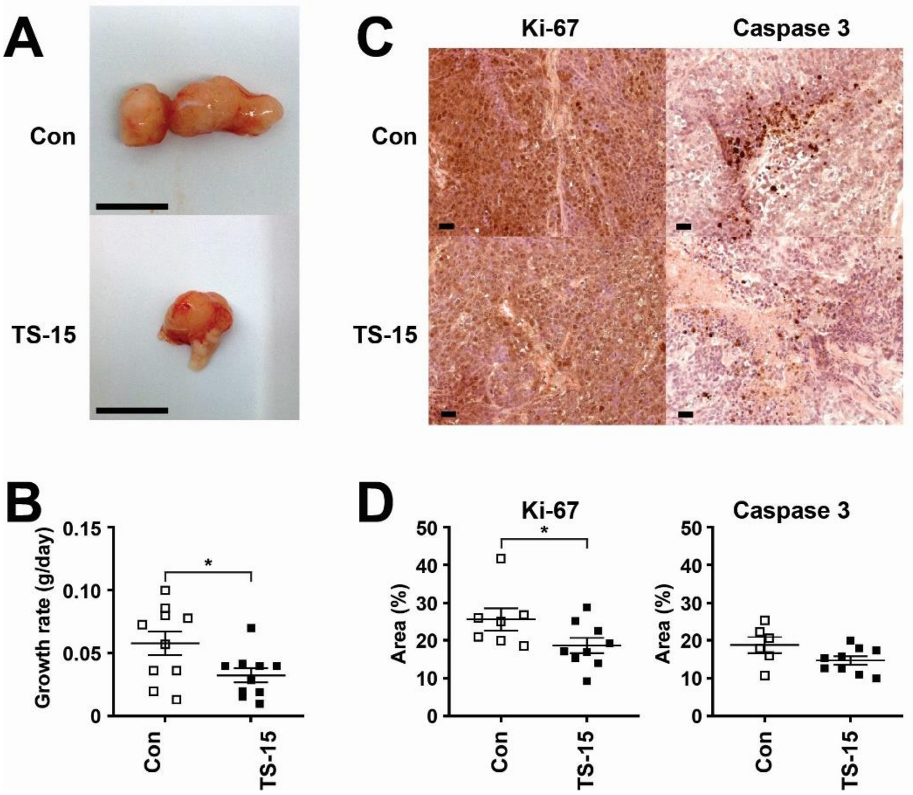

Supp. Figure 2. Impact of ADAMTS-15 expression on tumor growth, proliferation and apoptosis. **A-D.** Tumors harvested from intact mice injected with control (Con) and ADMATS-15-expressing (TS-15) LNCaP cells were imaged (**A**), weighed to determine relative growth rate (**B**), and sectioned for immunostaining with for Ki-67 and Caspase for imaging (**C**) and quantification of relative area of staining (**D**). Images in panel **A** (scale bar = 0.5 cm) and **C** (scale bar = 10 μm) are from representative samples, while the graphs in panel **B** and **D** present results for tumors from individual mice, showing mean and S.E.M. (*$p<0.05$, n=6-11).
